# Promoting multi‐micronutrient powders (MNP) in Peru: acceptance by caregivers and role of health personnel

**DOI:** 10.1111/mcn.12217

**Published:** 2015-11-01

**Authors:** Hilary Creed‐Kanashiro, Rosario Bartolini, Melissa Abad, Varinia Arevalo

**Affiliations:** ^1^ Instituto de Investigación Nutricional Lima Peru

**Keywords:** multi‐micronutrient powders, anaemia, acceptability, health personnel, infant and young child feeding, Peru

## Abstract

Iron deficiency causes anaemia and other adverse effects on the nutritional status and development of millions of children. Multi‐micronutrient powders (MNP) have been shown to reduce anaemia in young children. In Peru, 50% of children 6–36 months are anaemic. Since 2009, the government has started distributing MNP. This qualitative study explored the acceptability of MNP by caregivers and the role of health personnel (HP) in three regions (Apurimac, Ayacucho and Cajamarca), piloting the MNP programme between 2009 and 2011. Data collection consisted of interviews (35) and observations (13) with caregivers and HP (11). In Cajamarca, 16 families were visited three times in their homes to understand caregivers' use and difficulties. Results showed the critical role HP has in influencing caregiver understanding and use of the MNP, as well as the need for training to avoid confusing messages and provide counselling techniques that consider cultural sensitivity to optimize HP interactions with caregivers and adapt the recommendations for MNP use to local family feeding routines. There was greater acceptance of MNP by caregivers giving semi‐solid foods (e.g. purees) to their children than those who served dilute preparations (e.g. soups). Acceptance was similar across regions, but there were some differences between urban and rural settings. Home visits were shown to be a key in improving the use of MNP by caregivers as misunderstandings on preparation, required consistency and optimum practices were common. These findings can contribute to strategies to enhance acceptability and use.

**Key messages:**

- Acceptance and use of multi‐micronutrient powders (MNP) by caregivers greatly depend upon how it is presented, promoted and counselled by health personnel.
- Counselling for MNP use needs to consider and adapt to the local cultural context and incorporate family and child feeding routines.
- MNP are presented as part of appropriate feeding practices, encouraging caregivers to find simple and acceptable ways of giving semi‐solid or solid foods with which to mix it.

© 2015 Blackwell Publishing Ltd

## Introduction

Micronutrient deficiencies and especially iron deficiency continue to be a problem in many parts of the developing world causing anaemia and other adverse effects on the nutritional status and development of millions of children. Strategies to address these deficiencies include supplementation, fortification and improving dietary quality. The latter is often limited in deprived settings because of poor access to micronutrient rich foods, especially animal source foods (Dewey & Vitta 2013). Thus, iron supplementation and home fortification through the use of multi‐micronutrient powders (MNP) or pre‐fortified products such as lipid nutrient supplements have been used. Although these strategies have been shown to be efficacious when consumed by children (Dewey *et al*. 2009; Zavaleta *et al*. 2013), their success ultimately depends upon the acceptance and appropriate use by the target populations.

Multi‐micronutrient powders have been shown to reduce anaemia in infants and young children from 6 months to 2 years of age in efficacy trials in Ghana (Zlotkin *et al*. 2001; Adu‐Afarwuah *et al*. 2008) and Cambodia (Giovannini *et al*. 2006), as well as in programmatic settings in Bangladesh (Ip *et al*. 2007) and Haiti (Menon *et al*. 2007). However, acceptance and appropriate use of MNP by target populations have ranged from 30–43% among low‐income families in the United States (Geltman *et al*. 2009) to 60% in Inuit children of Canada (Christofides *et al*. 2005) and were very variable in refugee and emergency situations in Bangladesh, Nepal and Kenya (Rah *et al*. 2012), although higher acceptance has been found in smaller more controlled situations (Ip *et al*. 2007; Adu‐Afarwuah *et al*. 2008; Loechl *et al*. 2009; Jefferds *et al*. 2010).

In Peru, 50% of children 6–36 months are anaemic (INEI 2011, INEI 2014). Since 2009, the Peruvian government has implemented a pilot programme for the distribution of MNP (named ‘Chispitas’) to replace the current norm of supplementation with ferrous sulfate (liquid). The pilot was carried out in four regions, to reach a total of 100 000 children. The MNP contain five micronutrients: iron, zinc, folic acid, vitamin A and vitamin C. A midterm evaluation of this pilot programme in the region of Apurimac indicated there was variability in the acceptance and use by caregivers, and the prevalence of anaemia in the population had not diminished (Huamán‐Espino *et al*. 2012).

Due to the inadequate consumption of MNP and lack of impact on anaemia in the target population, the present study was designed to explore and understand the acceptability and use of MNP among caregivers and health personnel (HP) in order to identify strategies to enhance its use by caregivers.

## Methodology

This was a qualitative study conducted in two phases across three regions.

*Phase 1* consisted of interviews and observations with caregivers of children 6–36 months in their homes and HP to explore issues of acceptability. It was conducted in Ayacucho and Apurimac, where the pilot MNP programme had been implemented and the prevalence of anaemia was 52.9% and 61.9%, respectively (ENDES 2011). The pilot programme had started in 2009; the present study was conducted during September–October 2010.

*Phase 2* comprised interviews and observations of counselling sessions in the health facilities (HF), and follow‐up interviews, visits and observations of caregivers using MNP in their homes. It was conducted in the region of Cajamarca – anaemia prevalence 59.8% (ENDES 2011) where the distribution of MNP was just starting. The programme in Cajamarca started late 2010; this study was conducted during February–March 2011.

### The ministry of health MNP distribution regime

The MNP were distributed freely to caregivers of children 6–36 months through the local HF at their monthly (or bi‐monthly for children >1 year) *Control de Crecimiento y Desarrollo* (CRED) – growth and development monitoring consultation. The explanation regarding MNP and its use is given by HP. At the time of this study, MNP sachets were distributed to be given to the child every other day for 6 months. This was followed by a rest period of 6 months before starting the next 6‐month round (MINSA& MINDES Ministerio de Salud (MINSA) &Ministerio de la Mujer (MINDES), Peru 2009).

#### Phase 1 Ayacucho and Apurimac

In each region, a peri‐urban and a rural HF were selected for diversity. At each HF, caregivers with children 6–36 months who were receiving or had received MNP participated in the study, located through the HF or by other caregivers. HP involved in or responsible for the MNP distribution programme were interviewed. Observations were made during CRED consultations and at home when the caregivers prepared food.

In‐depth interviews were conducted with participants using semi structured guides. Topics explored with HP included knowledge of MNP, the incorporation of the programme into their daily activities and information and communication with caregivers. Topics explored with caregivers included level of understanding and perceptions of MNP, their use and benefits, explanations received from HP and their experience of using them.

#### Phase 2 Cajamarca

In Cajamarca, three rural HFs where the MNP programme had recently started were selected for the study. Caregivers who had just begun receiving MNP were included in the study, located using similar methods as in Phase 1. In‐depth interviews were conducted with HP involved in or responsible for the MNP programme, and observations were made of the CRED consultations when MNP were delivered to the caregiver.

Caregivers receiving MNP were visited subsequently in their homes on three occasions, following the methodology of household trials (PAHO &UNICEF, Pan‐American Health Organization (PAHO) & UNICEF 2013). At the first visit, the preparation and giving of the child's food with MNP were observed, followed by an in‐depth interview. As in household trials, motivational messages and recommendations for use, based on the current Ministry of Health indications, were discussed (benefits of MNP, preparation of MNP with thick food and adding MNP to two tablespoons of food to ensure he/she eats it all) and in addition discussing with the caregiver alternative foods, portion sizes, times and convenience of giving. Caregivers were visited the second time (between 3–5 days), and on the third occasion (days 7–8) when further observations and interviews were conducted, difficulties and alternatives were discussed at each visit.

### Data analysis

The recorded interviews were transcribed and coded by themes. These were organized into thematic matrixes by region, type of participant and by peri‐urban and rural areas. The four researchers read all the matrixes, noting coincidences and differences within each region and between regions for each theme. The principal findings and comprehension of the data were discussed and consolidated.

For the household follow‐up in Cajamarca, matrices were constructed for each case to follow the complete sequence of visits to understand the process for each participant. The same strategy as described previously was followed with discussions of each case among the four researchers to arrive at the principle findings and note the similarities and differences between cases.

### Ethics

The study was approved by the Research Ethics Committee of the Instituto de Investigación Nutricional. All participants interviewed or observed gave their verbal consent prior to each study activity.

## Results

The number of interviews and observations conducted by site is shown in Table 1.

**Table 1:** Number of interviews and observations conducted by site

Health facility	Ayacucho			Apurimac			Cajamarca, rural			
	Santa Elena, peri‐urban	Huamanguilla, rural	Total	Centenario, peri‐urban	Ccoc‐hua, rural*	Total	Mikuypampa	El Mangle	La Encañada	Total
Caregivers: interviews	7	9	16	9	10	19				
Caregivers, observations in the home	4	2	6	4	3	7				
HP interviews	2	2	4	2	2	4	1	1	1	3
Observations in CRED	1	1	2	2	2	4	2	3		5
Home follow‐up caregivers							2	3	11	16

HP, health personnel; CRED, Control de Crecimiento y Desarrollo.

* Five interviews with caregivers were conducted with the aid of a translator (a female community member) as caregivers conversed in Quechua.

### Experience and perspectives of HP

Health personnel in the study sites had received one training session on MNP distribution that focused on the following:

- nutrient (especially iron) content of the MNP and its characteristics,
- importance to prevent anaemia and promote growth,
- dosage and frequency of giving,
- preparation: mixing with two spoonful of the child's food at lunch,
- consistency and temperature of the food with which to mix it,
- absence of side/harmful effects and
- use during illness (conflicting information).

There was generally no attention to optimum counselling techniques and cultural adaptation of the messages and use of MNP in the context of the home environment. Also, not all HP had received the same training that was by the ‘cascade’ methodology, with information being modified as it was transferred at each level.

The principal facilitating factors and barriers to using the MNP resulting from the analysis of the interviews with the HP are shown in Table 2. HP mostly understood the composition, benefits and use of the MNP, to improve growth and development of the child and to prevent anaemia. They had a favourable opinion, recognizing that MNP ‘complement diets which are inadequate in animal foods’ (*HP*, *Apurimac*).

Overall, HP showed adequate knowledge of how to use the MNP, with semi‐solid complementary foods: ‘…are absorbed better in solid food, in the liquids they float and are not well dissolved’ (*HP*, *Ayacucho*). Thus, promoting MNP was providing an opportunity to focus on appropriate feeding practices. However, there were some misconceptions, such as: ‘they shouldn't be mixed with egg or milk as these already have vitamins’ (*HP*, *Cajamarca*). Doubts expressed were in relation to the temperature of the food and whether to give when the child was ill. They had tasted the sprinkles and did not find them disagreeable, although one nurse mentioned that ‘it leaves something in the mouth’ (*HP*, *Ayacucho*).

However, a major limitation of HP was how the MNP were promoted to caregivers (Table 2). Observations of the CRED counselling showed variation in terms of content and advice given, generally vertical in style (i.e. giving instructions/orders of what the caregiver should do, no negotiation of possibilities or feasibility), and focused primarily on how MNP should be prepared and given to the child. Motivational or culturally appropriate counselling for behaviour change was mostly not applied. However, some HP reported their cultural adaption of the messages, e.g. ‘the Chispitas increase the blood in your child’ (*HP*, *Apurimac*), ‘your child will have bright eyes’ (*HP*, *Apurimac*) and ‘…above all for child's intelligence’ (*HP*, *Cajamarca*). Recommendations of the time of giving were also adapted: ‘In our health center we decided that a good strategy would be to give it at breakfast, at 6am in a spoonful of purée, when mothers say their children are most hungry…’ (*HP*, *Apurimac*).

Observations in the HF showed that none of the specific educational materials were used with the exception of a pamphlet in Ayacucho and posters in some rural areas. ‘I may have some educational material but I cannot say where it is’, (*HP*, *Cajamarca*). Several HP mentioned that certain print materials were not very useful, especially for the rural, Quechua‐speaking population whereas the radio and television campaigns were considered helpful in facilitating MNP introduction through the HF.

Health personnel reported that caregivers had commented positively about the MNP. ‘My child's appetite has increased’ (*HP*, *Cajamarca*) and ‘my child asks for: “my sachet, my food” ’, (*HP*, *Ayacucho*). However, they mentioned that some mothers did not want to use them because of doubts regarding the benefits: ‘as it is donated we do not know what is in it; it may make our children brutish’ (*HP*, *Ayacucho*) and a few reported that it was bothersome to prepare. Other concerns expressed frequently by mothers to the HP were related to change in the taste of the food, subsequent diarrhoea and what to do if they forget to give them.

**Table 2:** Principal barriers and facilitating factors to using MNP. Analysis of the information regarding health personnel

Limiting factors	Facilitating factors
Focus on the ‘how’ to prepare and give it, caregivers require more information on what and why.	HP recognizes anaemia is a problem; MNP is an appropriate strategy. Appreciation of benefits to child.
Confusion on part of HP, e.g. what types of preparations to mix MNP with including sometimes recommending soups, liquids and dilute foods. How much food to mix with.	Many HP have knowledge of what to mix with and how to prepare it.
Contradictory messages.	
Confusion about what foods it should not be mixed with, e.g. eggs and liver, as these are ‘high in nutrients and can interfere with MNP’.	Some HP have innovative ideas, e.g. mix with banana.
Uncertainty about what to do when the child is ill (give ferrous sulfate when ill).	Current MOH norms clarify this. Recognized advantages over ferrous sulfate supplements due to absence of side effects, ease of giving to child.
‘Poor’, vertical counselling on what caregiver must do. Little attention to cultural situations, perceptions, routines and practices.	Some HP adapt the messages to local culture, caregivers' motivations and seek feasible strategies of when and how to use it with caregivers, e.g. ‘when the child is hungry’.
Lack of (and inappropriate) education materials and tools.	Radio and TV campaign support HF messages.
Some HP have not tasted MNP or consider it has poor taste.	Mention that taste is not detectable.
Currently, too many feeding recommendations/messages given at one counselling session.	Attention on nutrition in CRED counselling, promotion of thick complementary foods and use of growth curve.
Infrequency of demonstration sessions.	Use of demonstration sessions to facilitate learning.
Lack of follow‐up with home visits, lack of coordination with community/health promoters, other programmes.	Coordination with health promoters or other local organizations to support home visits.
How to address a situation when caregivers forget to give MNP.	Current programme is daily dose.
Periods of non‐availability to HF.	Currently available continuously.
The 6‐month ‘rest period’ with no MNP, thus infants did not receive it at critical ages.	

MNP, multi‐micronutrient powders; HP, health personnel; TV, television; HF, health facilities; CRED, Control de Crecimiento y Desarrollo; MOH, Ministry of Health.

### Caregivers' experience and perceptions

The principal facilitating factors and barriers to using MNP by caregivers are shown in Table 3. The predominant recommendation recalled by caregivers at all sites was how to prepare MNP and give it to their children, rather than what they were or why. They mostly remembered that HP told them to mix it in thick food and to give it every other day. However, some misconceptions were identified, e.g. they reported being told to give them in soups, or not to give with liver or eggs, ‘as they can interfere with MNP’ (*CG*, *Cajamarca*).

Caregivers reported differences in their experiences of giving MNP. Most reported mixing it with thick savoury foods, others with soups or milk. Only in Cajamarca did they mention that they also mixed it with fruit such as mashed banana, a helpful recommendation they received from HP.

‘I do better with banana because he eats more; with potato he hardly eats any, it must be because it has a taste.’ (*CG Cajamarca*).

‘I gave it to him in his pudding, before I hadn't given it. I gave it because I was in a hurry, I thought he would not eat it but he did. But he likes it better with fruit than with the lunch or pudding.’ (CG Cajamarca).

Several caregivers mentioned that their children disliked food with MNP, particularly those who mixed it with soups or other liquids. Others mentioned their child disliked it at first, but they had insisted with small amounts or mixed in the MNP when the child was not looking. Most caregivers mixed MNP with two spoonful of food, but there was a wide variation from 1–6 spoonful to a whole plateful, especially in Cajamarca, thus limiting the dose the child received when he/she did not finish it. ‘This amount (..in 2 spoonsful..) is better for my child, because he can eat this much. It will not do him any good if it is mixed with a lot, it'd be like we haven't given him any, he wouldn't finish it.’ (*CG Cajamarca*). Generally, caregivers found that the MNP had neither taste nor smell. Some mentioned black specs that were disagreeable (particularly when mixed with liquids), but others mentioned that these disappeared when mixed with thick food and were not noticeable when mixed with mashed banana.

Most of the caregivers did not know what the MNP were, only a few mentioned vitamins or iron, but they did say they are to prevent anaemia, ‘strengthen the blood’; to increase the child's appetite; to increase weight and height and develop their intelligence, and ‘mentality’; ‘to grow and vitamins for her brain’, (*CG*, *Ayacucho*); ‘to have more appetite, it has iron for her bones, vitamins for her brain’, (*CG*, *Ayacucho*) and ‘I don't remember what she said but it has many benefits’, (*CG*, *Ayacucho*).

In contrast, some peri‐urban caregivers in Ayacucho and Apurimac were concerned that the MNP might harm their child, causing diarrhoea and vomiting or making the child retarded. Concerns mentioned included the following: what is in the sachet?, What is it for?, Is it given to others or only to me? and Will it harm my baby, especially babies aged 6 months? The majority of these caregivers eventually tried giving it to their child after receiving reassurance from a relative or neighbour supporting the idea that they are vitamins. However, some mothers had received negative comments from others making them suspicious of the product: that they are to make the children brutish, or they are to sterilize the girls. ‘They come from another country so that they take my child away. That is why I give them to my pigs and dogs’ (*CG*, *Ayacucho*).

Several benefits were perceived by the caregivers as a result of giving MNP to their children, including noting increased appetite, weight and growth, less diarrhoea and improved colour of the child. In contrast, some caregivers mentioned constipation, vomiting and diarrhoea among negative effects, as well as a concern that in spite of taking MNP, there are children who have not recovered from anaemia.

Nevertheless, caregivers expressed preference for MNP to ferrous sulfate, not only for the taste and ease to give to the child but also to keep, transport and because ‘with MNP you are also giving food, so the child eats’ (*CG*, *Ayacucho*).

**Table 3:** Principal barriers and facilitating factors to using of MNP. Analysis of the information regarding caregivers

Limiting factors	Facilitating factors
Concerns of whether it could harm the child.	Recognize the benefits of giving MNP. Observe benefits in their children, growth, development, and increases appetite. Confidence in HP.
	Prior knowledge of benefits of vitamins.
	MNP are perceived as vitamins and good.
Confusion on how to prepare it, confusing indications from HP and misunderstandings.	Foods of appropriate consistency most frequently used. Mix with the food that the child will eat.
Custom to serve plate of food to child and MNP mixed with all and the child does not finish.	Mix in same plate or in separate plate and then give the rest of food.
	Recognition that child needs to consume the portion of food with MNP.
Custom to give dilute foods, reluctance to change and some children do not accept thick foods.	Understanding that they are not as effective or acceptable when mixed with dilute/liquid preparations.
	Versatility in food preparations and inclusion of sweet foods such as fruits and banana.
Caregivers say the child does not like the food with MNP.	Most children eat the food with MNP; some ‘ask’ for their sachet. Some caregivers mix MNP into food when the child is not looking.
Some mothers have not tasted the MNP.	Advantages over ferrous sulfate.
Attribute negative acceptance/illness of the child to MNP.	Ease of preparation and convenience of storage.
Forgetting to give as every other day and not knowing what to do.	Beneficial home visits especially with support of other local institutions.
Withholds when the child is ill.	Gives when the child is most hungry, e.g. breakfast and main meal.
Receive negative opinions from others.	Receive positive reinforcement from family and others.
	Mothers with good experience share the positive messages.
	Participation in demonstration sessions to prepare and taste with local foods.
	Radio and TV campaigns.

MNP, multi‐micronutrient powders; HP, health personnel; TV, television.

### Follow‐up of caregivers in Cajamarca

Fourteen of the 16 caregivers followed up in their homes after receiving MNP from the HF completed the three visits. At the initial home visit, all 16 were giving MNP to their child, but only three mothers were giving it according to indications. Five mothers were observed to mix the MNP in soups or milk, and eight mixed it with food of a thick consistency but mixed into the full serving (instead of the recommended two spoonful).

Table 4 summarizes the observed changes in practices during the household trial and the reasons why some families had not adopted the recommendations at follow‐up.

**Table 4:** Observed changes in practices and barriers and facilitators to behaviour change, follow‐up in home, Cajamarca

Recommendation	Number of caregivers following recommendations		Reasons for not changing practice
	Initial visit	Final visit*	
Offer MNP mixed in thick foods.	9/16	12/14	Child refused solid foods,
			Continued to give with soups/milk,
			Child disliked food with MNP,
			Not given during diarrhoea,
			Misunderstanding of recommendation of which foods to mix with from health facility.
Mix MNP in two spoonful of food.	3/16	9/14	Did not separate two spoonful: usual routine of serving plateful; remembered the recommendation but continued to serve in the whole portion.

MNP, multi‐micronutrient powders.

* A total of 14 completed the follow‐up visits.

The principal limitations to adoption were related to the following cultural practices: (i) giving dilute complementary foods instead of semi‐solid or solid foods; (ii) serving a plate of food to the child and not separating out a portion to mix with the MNP and (iii) receiving confusing messages from HP regarding what to mix it with and what to do when child is ill. Mixing the MNP with fruit (e.g. mashed banana) was favoured.

## Variability across regions and rural‐peri‐urban sites

### Health personnel

Knowledge of MNP by HP was similar across all regions as were the doubts and concerns. In all regions, HP had difficulties in explaining MNP to caregivers. However, in Apurimac, there were more complementary food demonstrations using MNP and promotion of nutritious foods as well as household visits, in part due to the activity of the non‐governmental organization (NGO) CARE Peru, collaborating with the local Ministry of Health.

There were mostly similarities but also some differences between peri‐urban and rural HP. More doubts around MNP were expressed by HP in the peri‐urban settings, such as whether to give MNP or ferrous sulfate if anaemia is diagnosed, but there was also more emphasis given on its preparation and the time of giving in the home. HP in peri‐urban settings reported that the reasons caregivers did not give the MNP were because the child did not like it; it made the child sick, and the caregiver had doubts about it. In rural areas, the reasons were attributed to the caregiver forgetting to give it or that they do not recognize the value of the MNP.

### Caregivers

The information that was most remembered by caregivers was similar across regions and urban and rural areas, that MNP were to be mixed with foods of thick consistency, and as part of the main course at lunch. But also, some caregivers in all regions and both rural and peri‐urban understood that the MNP can be mixed with dilute preparations. ‘You give it mixed into potato, also main course and soups.’ (*CG rural*, *Apurimac*).

‘You have to mix it with some food, in soup or main course.’ (*CG urban*, *Apurimac*).

However, there is obviously a difference in understanding of ‘thick consistency’ between HP and caregivers as illustrated in the following citations.

‘Well mashed, with potato or pea flour soup. That's what she told me, in pea flour soup with egg. Also with noodle soup, but well mashed. I have also given it with semolina soup.’ (*CG rural Cajamarca*).

‘They told me to give him a little, in soups. Just in soups they told me. Thick soups, because you shouldn't give in water. It must be because it could spill or perhaps he won't want it.’ (*CG rural Cajamarca*).

The motivations for giving the MNP in all three regions and across rural and peri‐urban settings were the benefits for the child, related to growth and development (strong and intelligent) and thus for appetite, protection against ill health and ‘Good for the blood’.

In Cajamarca (rural) and rural Ayacucho, caregivers added the positive motivation that it was recommended by HP. ‘I thought that they know what to give, what is good for the children, that's why I gave it.’ (*CG rural Ayacucho*).

‘Vitamins … so she is happy…to grow. I gave them because they told me to give them.’ (CG rural Cajamarca).

More mothers reported stopping giving them in the peri‐urban areas, and for different reasons than in rural areas, similar to those mentioned previously by HP. ‘He rejected it’ (*CG urban Ayacucho*).

‘He did not want to eat, he spat it out.’ (CG urban Ayacucho).

The practices were similar across regions and settings as described previously. However, MNP were mixed with fruit in Cajamarca, as a result of HP recommendations, and found to be more successful; this was not observed elsewhere. In rural areas, it was common for caregivers not to express their doubts about the MNP during the CRED consultation, but home visits such as those carried out in Cajamarca by the research team and the NGO PREDECI provided a more comfortable opportunity for discussion.

## Discussion

The results of this qualitative study show that acceptability and use of MNP among the caregivers and their promotion by HP were variable; consequently, improvements in the programme are required in order to achieve a wider and more consistent use of the MNP.

This study indicates that there was greater acceptance and use of MNP by caregivers where HP appropriately adapted their interaction, rapport and counselling with caregivers to the local culture, taking time to understand her situation, explaining more about the MNP to absolve their doubts and inserting their use into the family and child's feeding routines, results similar to the findings of Young *et al*. (2010) and Jefferds *et al*. (2010). This cultural sensitivity and adaptation was observed to be uncommon in the counselling sessions and indicates the need for these issues to be addressed more specifically in the HP training. Caregivers said they did not feel comfortable voicing their concerns in the CRED consultation. Building a better rapport by HP to allow caregivers to discuss their concerns as well as providing correct and motivational information and adapting messages to local cultural practices and feeding routines in the home could reduce confusion and improve acceptance and use.

Nevertheless, an interesting aspect observed was the enhancement of the nutrition component in the CRED consultations; appropriate feeding practices were discussed with the mother usually before the topic of MNP was addressed, thus inserting MNP into an integral approach towards child feeding and nutrition and not presenting them as an isolated strategy.

There was greater acceptance of the MNP by caregivers who were accustomed to feeding semi‐solid or solid foods, whereas caregivers, who customarily served dilute or liquid foods and added the MNP to these preparations, showed less acceptance and often discontinued giving it to their children. This was due to disagreeable taste, failure to dissolve and being more noticeable in the food or drink given. Acceptance was related to how the child had been fed prior to receiving the MNP. Several caregivers were able to change this practice with the home follow‐up visits and encouragement in Cajamarca.

The considerable variation in how MNP was prepared and the foods and amounts with which they were mixed and given indicate the need to specifically address preparation practices to ensure the child receives an optimum dose. This includes addressing the ‘separate two spoonful of the child's food’ to mix with the MNP, and adapting this to practices that the caregiver finds most acceptable. Offering feasible alternatives, for example, mixing with fruit (mashed banana), giving MNP ‘when the child is most hungry’ and at the first meal of the day, were successful strategies.

Both HP and caregivers were aware of the benefits of MNP. Most caregivers were motivated to use them, citing the perceived health benefits, their confidence in the local HP (although this varied according to level of urbanization) and how the child accepted MNP. Caregivers had general knowledge about anaemia and that vitamins are valued for healthy growth, thus facilitating introduction as reported elsewhere (Geltman *et al*. 2009; Jefferds *et al*. 2010). Where doubts regarding the product and its use were expressed, caregivers were influenced by relatives and neighbours (Geltman *et al*. 2009), mostly positively but also negatively. The benefits perceived by mothers as a result of giving MNP to their children were positive and similar to benefits reported in Ghana (Adu‐Afarwuah *et al*. 2008) and in Niger (Tripp *et al*. 2011). In turn, these benefits were promoted and reinforced by some HP. As in Haiti (Loechl *et al*. 2009), preparing caregivers with a radio and television campaign prior to the distribution of MNP was found to increase acceptance. Similar to that reported in Ghana (Adu‐Afarwuah *et al*. 2008), both HP and caregivers found that the MNP had considerable advantages over ferrous sulfate syrup (Flores *et al*. 2008) or drops, due to the lack of side effects, ease of use (Young *et al*. 2010) and its association with food.

Most of the messages, practices and concerns, although varied, were similar across regions although there were some notable differences, such as the practice of mixing MNP with fruit in Cajamarca. Support from local NGOs also facilitated some HP activities. Differences between peri‐urban and rural settings tended to relate to the types of concerns of HP, reflected in the caregivers, and reasons to stop or not use MNP. The differences observed in developing rapport depended more on personality and commitment of the HP rather than health service structure and training. The cultural sensitivity and adaptation shown by the HP in rural Apurimac are an example of this personal initiative.

Home visits, such as the household follow‐ups in Cajamarca by the research team, were found to be very beneficial in helping caregivers to incorporate the MNP appropriately, providing an opportunity to discuss misconceptions and unclear advice received from the HF, explore with caregivers their daily usual feeding practices and resolve concerns and queries. This was particularly important as caregivers stated that they did not feel comfortable voicing their concerns in the CRED consultation. Complementary feeding demonstrations and home visits are part of routine functions of HP, but they do not occur with the established frequency. In Cajamarca follow‐up, home visits were made by the supporting NGO PREDECI, and this led to enhanced understanding and use of MNP in the homes indicating that this strategy can greatly facilitate MNP acceptance and use. These additional activities have been reported as helpful components in Nigeria (Tripp *et al*. 2011), Haiti (Loechl *et al*. 2009) and Kenya (Jefferds *et al*. 2010).

In‐depth formative research including the methodology of household trials is necessary to inform and develop appropriate strategies to deliver interventions for a specific population (PAHO, UNICEF Pan‐American Health Organization (PAHO) & UNICEF 2013) although it does not necessarily guarantee acceptance on a wider scale. It has been used successfully to explore acceptance and develop interventions for MNP in Timor‐Leste (Osei *et al*. 2014), Niger (Tripp *et al*. 2011), Kenya (Jefferds *et al*. 2010) and Mexico (Young *et al*. 2010). In this study, we have used the methodology to explore issues arising in an ongoing programme. The use of household trials in Cajamarca where the introduction of MNP was just starting helped us to understand initial programmatic problems and showed the advantages of home visits.

The results of this study indicate that (i) a greater understanding of the MNP on the part of the caregiver; (ii) the motivation of observing their child have a ‘bigger appetite’ is ‘brighter’ and (iii) learning how to use it, as in the demonstrations in Apurimac and in the home in Cajamarca, all contributed to increased acceptability and use. Typically, HP advice focused on giving verbal instructions on how to use MNP in the home, but this is insufficient to motivate the caregiver to use it.

As a result of this study, the following recommendations to increase acceptability and use of MNP by caregivers and which can be incorporated into the national scaling up of the programme are suggested:

- Improve and extend the training of HP with regard to MNP, to ensure correct understanding and use and avoid confusions. Improve cultural sensitivity and counselling techniques of HP with caregivers. These include giving the necessary information, negotiating with caregivers the incorporation of MNP into their daily feeding practices considering the local cultural context, use simple, culturally appropriate pictorial educational materials during counselling and invite the mothers to taste the MNP when they are first introduced to them.
- Ensure all HP in a health facility in contact with caregivers give the same messages regarding MNP and that all children 6–36 months receive it, not only during CRED consultations.
- Assure follow‐up to caregivers in their homes during the first weeks and months to discuss their concerns, help them resolve problems and support sustained use.
- Help caregivers find simple alternatives of local foods and food preparations with which to mix the MNP, ensuring the appropriate consistency, to increase acceptance.
- Conduct mass media communication campaigns and incorporate local authorities, organizations, community leaders and other influential leaders of opinion and decision‐makers in the process of positioning MNP.

There are several limitations to this study, specifically the small sample both of the intervention areas and the number of HP and caregivers interviewed, observed and followed. In addition, the selection process of caregivers from the health services or in the community could have led to a bias towards families who were using MNP and thus had a favourable attitude. Other family members were not included, so their perspective has not been considered directly, although some caregivers commented on the opinion of their husband or relatives. In some areas of Ayacucho and Apurimac, the study was conducted at a time when MNP were not available; thus, opportunities of observing its use in the HF and home were limited. As this was only a small sample, we were unable to determine acceptability of MNP by age of the child although there were more comments from mothers of older children that their child ‘did not like the food with MNP’. However, the acceptance was largely related to prior feeding patterns, particularly consistency; greater rejection was seen where the child was used to receiving dilute preparations, independent of his/her age. This is an area to be further explored.

This study focused on aspects of acceptability both by HP and caregivers, rather than aspects of MNP distribution. However, another evaluation of the programme in these regions (ACH & UNICEF Acción contra el Hambre (ACH) &UNICEF Peru 2013; UNICEF Perú 2014) indicated that mothers discontinued or interrupted the use of MNP when they did not attend the HF, in part due to cost and time, also that the supply of MNP at the health services was not always reliable, indicating that reduced use was not only due to less acceptability but also accessibility.

This study provides insights into the acceptability of MNP in selected highland areas in Peru and issues that can be addressed to encourage and broaden their use in this specific socio‐cultural context. Our results show many similarities to other acceptance studies in Peru (ACH, UNICEF, Acción contra el Hambre (ACH) &UNICEF Peru 2013) and in other parts of the developing world, regarding the benefits perceived with the use of MNP as well as the concerns and suspicions on the part of caregivers and their families, placing the inadequate use as a result of the caregivers' perceptions and behaviours. However, a unique feature of this study is linking the acceptability and use by caregivers to the health services at the local level, learning of the limitations from the perspective and actions of HP and examining ways to improve the MNP programme within the health services. Health service performance around MNP and its linkage with caregivers' use are an area for further research to enable increased success in programmes to reduce anaemia in children.

The MNP programme in Peru has recently extended to several other regions, and a change in regimen to a daily dose has been implemented. The results presented here of specific issues that need to be addressed to ensure children receive the required dose in their home as well as the methodologies used to elucidate these issues can be of use both in Peru and for other countries implementing similar strategies to reduce micronutrient deficiencies in infants and young children.

## Conflicts of interest

The authors declare that they have no conflicts of interest.

## Contributions

H. C. K. and R. B. were responsible for the design of the study and training for data collection in the field, implementation and supervision, and analysis and writing the manuscript. M. A. and V. A. conducted the data collection in all three sites, prepared the field reports and constructed the data into the analysis matrixes. All four authors contributed to the manuscript.
